# The prostate metastasis suppressor gene NDRG1 differentially regulates cell motility and invasion

**DOI:** 10.1002/1878-0261.12059

**Published:** 2017-05-02

**Authors:** Anup Sharma, Janet Mendonca, James Ying, Hea‐Soo Kim, James E. Verdone, Jelani C. Zarif, Michael Carducci, Hans Hammers, Kenneth J. Pienta, Sushant Kachhap

**Affiliations:** ^1^ Prostate Cancer Program Department of Oncology The Sidney Kimmel Comprehensive Cancer Center Johns Hopkins Medical Institutions Baltimore MD USA; ^2^ Department of Urology The James Buchanan Brady Urological Institute The Johns Hopkins University Baltimore MD USA

**Keywords:** actin cytoskeleton, matrix metalloproteases, metastasis, prostate cancer

## Abstract

Experimental and clinical evidence suggests that *N‐myc downregulated gene 1* (*NDRG1*) functions as a suppressor of prostate cancer metastasis. Elucidating pathways that drive survival and invasiveness of *NDRG1*‐deficient prostate cancer cells can help in designing therapeutics to target metastatic prostate cancer cells. However, the molecular mechanisms that lead *NDRG1*‐deficient prostate cancer cells to increased invasiveness remain largely unknown. In this study, we demonstrate that *NDRG1*‐deficient prostate tumors have decreased integrin expression and reduced cell adhesion and motility. Our data indicate that loss of NDRG1 differentially affects Rho GTPases. Specifically, there is a downregulation of active RhoA and Rac1 GTPases with a concomitant upregulation of active Cdc42 in *NDRG1*‐deficient cells. Live cell imaging using a fluorescent sensor that binds to polymerized actin revealed that *NDRG1*‐deficient cells have restricted actin dynamics, thereby affecting cell migration. These cellular and molecular characteristics are in sharp contrast to what is expected after loss of a metastasis suppressor. We further demonstrate that *NDRG1*‐deficient cells have increased resistance to anoikis and increased invasiveness which is independent of its elevated Cdc42 activity. Furthermore, NDRG1 regulates expression and glycosylation of EMMPRIN, a master regulator of matrix metalloproteases. NDRG1 deficiency leads to an increase in EMMPRIN expression with a concomitant increase in matrix metalloproteases and thus invadopodial activity. Using a three‐dimensional invasion assay and an *in vivo* metastasis assay for human prostate xenografts, we demonstrate that *NDRG1*‐deficient prostate cancer cells exhibit a collective invasion phenotype and are highly invasive. Thus, our findings provide novel insights suggesting that loss of NDRG1 leads to a decrease in actin‐mediated cellular motility but an increase in cellular invasion, resulting in increased tumor dissemination which positively impacts metastatic outcome.

**Abbreviations**
BSA: bovine serum albumin
ECM: extracellular matrix
EMMPRIN: extracellular matrix metalloproteinase inducer
FITC: fluorescein isothiocyanate
MMP: matrix metalloproteases
MTT: 3‐(4,5‐dimethylthiazol‐2‐yl)‐2,5‐diphenyltetrazolium bromide
NDRG1: N‐myc downregulated gene 1
PBS: phosphate‐buffered saline

## 1 Introduction

Preservation of tissue integrity is of prime importance to a multicellular organism; hence, multicellular organisms have devised barriers and mechanisms that prevent metastasis and safeguard tissue integrity. Cancer metastasis is a multistep process involving disengagement of tumor cells from the tissue of origin, intravasation into blood and lymph vessels, and extravasation to metastatic sites (Nguyen *et al*., 2009). Genes that hinder any one of these steps are likely to negatively impact the metastatic outcome. Such genes are termed as metastasis suppressors. Cancer cells functionally inactivate these metastasis suppressors through various mechanisms to conclude the process of metastasis (Smith and Theodorescu, 2009).

The well‐characterized Dunning AT6.1 spontaneous prostate cancer metastasis model aided in identification of *NDRG1* as a metastasis suppressor gene (Bandyopadhyay *et al*., 2003). Expression of NDRG1 inversely correlates with Gleason grading and overall prostate cancer patient survival (Bandyopadhyay *et al*., 2003; Liu *et al*., 2011; Song *et al*., 2010). In particular, NDRG1 expression in prostate tumors with lymph node or bone metastasis is significantly reduced as compared with those with localized prostate cancer (Bandyopadhyay *et al*., 2003). Decreased NDRG1 membranous expression correlates with prostate malignant progression and has significant negative impact on patient disease‐free survival. Besides prostate, NDRG1 protein is downregulated in breast, gastric, and colon cancer as well (Bandyopadhyay *et al*., 2004; Mao *et al*., 2013). Additionally, lower NDRG1 protein expression in the primary tumor predicts poor five‐year patient survival for all three tumor types (Bandyopadhyay *et al*., 2003, 2004; Liu *et al*., 2011; Mao *et al*., 2013). Although the clinical status of NDRG1 in these cancers is well documented, the exact molecular function of NDRG1 had remained elusive. Work from our laboratory revealed that NDRG1 can act as a Rab4a effector protein and help in endosomal transport of surface molecules including E‐cadherin (Kachhap *et al*., 2007a). In line with this, other groups have found that overexpression of NDRG1 inhibits epithelial–mesenchymal transition and beta‐catenin nuclear localization in prostate cells (Jin *et al*., 2014; Liu *et al*., 2011, 2012). Recently in a collaborative study, we demonstrated that NDRG1 could regulate centrosome homeostasis and genome stability (Croessmann *et al*., 2015). It is hypothesized that NDRG1 may serve as a multifunctional protein. Inherent in this postulate is the notion that NDRG1 may be capable of impacting cancer metastasis by more than one mechanism. However, the molecular mechanisms that lead to increased invasion in tumors that are deficient in NDRG1 remain poorly understood. In this study, we demonstrate that loss of NDRG1 causes differential activation of Rho GTPases and decreased actin dynamics. Surprisingly, loss of NDRG1 decreases cellular migration but increases matrix metalloproteinase (MMP) inducer EMMPRIN and MMP activity, leading to cellular invasion.

## 2 Materials and methods

### 2.1 Cell adhesion and spreading assay

Cells were plated at a density of 5 × 10^5^/mL in 60‐mm dishes, precoated with a proprietary extracellular matrix (ECM) mixture of fibronectin and collagen (FNC coating mix, AthenaES, Baltimore, MD, USA). Cellular adhesion was allowed to continue for 30 min at 37 °C, after which the plates were tapped to collect the nonadherent cells and counted. For cell spreading assay, cells were plated at 70% confluency on to a precoated coverslips with FNC coating mix. Cells were followed microscopically to visualize cell spreading and imaged every half an hour over a period of 6 h. Quantification of cell spreading was carried out using nikon tsi element software (Nikon Instruments Inc., Melville, NY, USA).

### 2.2 Cell migration and live cell imaging

Cell migration was assessed using standard scratch assay as described in previous reports (Nalla *et al*., 2010). Live cell imaging to assess cell migration was performed by plating cells on plates coated with FNC or Matrigel. Cells were allowed to adhere overnight and imaged using Nikon ECLIPSE Ti inverted research microscope (Nikon Instruments, Linthicum, MD, USA). Cells were visualized under phase‐contrast microscope and time‐lapse images were grabbed with a 10‐s interval for 5 min. Image sequences were exported as avi file for further analysis. To visualize actin dynamics, parental and knockdown cells were stably transfected with Lifeact‐RFP (kindly gifted by Dr. Robin Shaw, Addgene cat # 40908). Cells were plated on glass bottom dishes and visualized under live cell Zeiss LSM780‐FCS confocal microscope fitted with an environmental chamber. Images were grabbed using a 63X/1.4 PlanApo objective lens and exported as LSM files for further analysis. Kymograph analysis was performed using multiple kymograph plugin of imagej nih software (https://imagej.nih.gov/ij/). Kymograph readings were exported to Excel and plotted.

### 2.3 Rho, Rac, and CDc42 pull‐down assays

Cells were plated at a density of 2 × 10^6^ cells on 100‐mm dishes and allowed to grow for 24 hrs. On the day of the assay, cells were washed twice with ice‐cold PBS and resuspended in 800 μl of pull‐down lysis buffer for RhoA (50 mM Tris/HCl, pH 7.5, 1 mM EDTA, pH 8.0, 500 mM NaCl, 10 mM MgCl_2_, 1% (v/v) Triton X‐100, 0.5% sodium deoxycholate, 0.1% SDS, 10% (v/v) glycerol, and 0.5% (v/v) β‐mercaptoethanol), for Rac1 and Cdc42, supplemented with protease inhibitor cocktail (Roche), 25 mM NaF, 1 mM sodium orthovanadate, and 1 mM phenylmethyl sulfonyl (PMSF). Protein lysates were transferred to ice‐cold centrifuge tubes and clarified by centrifugation at 16 000 ***g*** for 20 min. A sample of the supernatant was saved for inputs. GST‐fused Rho‐binding domain (RBD) of rhotekin (kindly gifted by Dr. Martin Schwartz, Addgene Cat#15247) and p21‐binding domain (residues 67–150) of human PAK1 (kindly gifted by Dr. Jonathan Chernoff, Addgene Cat# 12217) bound to glutathione beads were used to pull down RhoA and Cdc42 and Rac1, respectively. Beads were added to the lysates and incubated at 4 °C with gentle rotation for 1 h. At the end of incubations, beads were washed with lysis buffer, suspended in Laemmli buffer, and heat‐denatured. Pull‐down proteins along with their respective inputs were separated on SDS/PAGE, electroblotted, and immunoprobed for respective proteins.

### 2.4 Confocal microscopy

Cells were plated on coverslips and allowed to grow to 70% confluence before being fixed, and permeabilized in 4% neutral buffered saline supplemented with 0.125% Triton X‐100 for 20 min at 37 °C. Permeabilized cells were blocked with 5% BSA in PBS for 2 h followed by overnight incubation with primary antibody. Cells were washed three times with 5% BSA in PBS followed by Alexa 488‐conjugated goat anti‐rabbit antibodies (Invitrogen). Actin was stained with rhodamine‐conjugated phalloidin (Molecular Probes) for 30 min at 37 °C. Nuclei were stained with DAPI (Sigma). Coverslips were mounted on slides and imaged using a Zeiss LSM510 Meta confocal microscope at the JHU microscopy facility. Images were processed using imagej software.

### 2.5 Gelatin zymography

Cells were maintained in respective serum‐free medium. Conditioned medium containing secreted MMPs was harvested by centrifugation at 800 ***g*** for 10 min and then stored at −70 °C. Proteolytic activity in the conditioned medium was analyzed by gelatin zymography in 0.1% gelatin/10% acrylamide gels. After development, MMPs were detected as transparent bands on a blue background, and the band area was quantitated with imagej nih software.

### 2.6 Invadopodia assay

Coverslips were coated with FITC‐conjugated gelatin matrix. FITC/gelatin‐coated coverslips were washed with RPMI containing 10% fetal bovine serum at room temperature for 30 min prior to plating cells. To assess the formation of invadopodia and degradation of FITC/gelatin matrix, cells were cultured for different time periods (8–16 h). Cells were fixed and stained for actin with rhodamine‐conjugated phalloidin. Cells were imaged using Zeiss LSM510 Meta confocal microscope, and matrix‐degraded area was quantitated using imagej software.

### 2.7 Three‐dimensional invasion assay

Three‐dimensional invasion assay was performed using protocols as described in our previous studies (Wissing *et al*., 2013). Briefly, cells were suspended in media containing 10% methylcellulose and grown as hanging drop for two days to form spheroids. For analysis of invasion, spheroids were embedded in either collagen or Matrigel matrices. Spheroids were imaged immediately after they were implanted in collagen matrix and following every day for 4–6 days. Invasion index was calculated as described previously (Wissing *et al*., 2013).

### 2.8 Prostate cancer metastasis assay

Metastasis assay for human prostate cancer cells was performed as described in our previous studies (Havens *et al*., 2008; Shiozawa *et al*., 2011). Briefly, PC3‐ and PC3‐*NDRG1*‐knockdown prostate cancer cell lines were trypsinized. Cells were counted and 2.0 × 10^6^ cells·mL^−1^ were mixed with Matrigel (1:1 volume). 1 × 10^6^ cells were injected subcutaneously in 10 four‐ to six‐week‐old NOD SCID male mice per group. Tumors were allowed to develop for three weeks. Animals were then sacrificed and tibias were harvested and stored at −80 °C until genomic DNA extraction. The number of disseminated cells was quantified using QPCR as described (Alcoser *et al*., 2011; Havens *et al*., 2008). Normalization was performed by quantitating mouse cells in each sample using primers specific to mouse *PTGER2* gene.

## 3 Results

### 3.1 NDRG1 deficiency results in decreased cell adhesion and spreading on ECM

**Figure 1:**
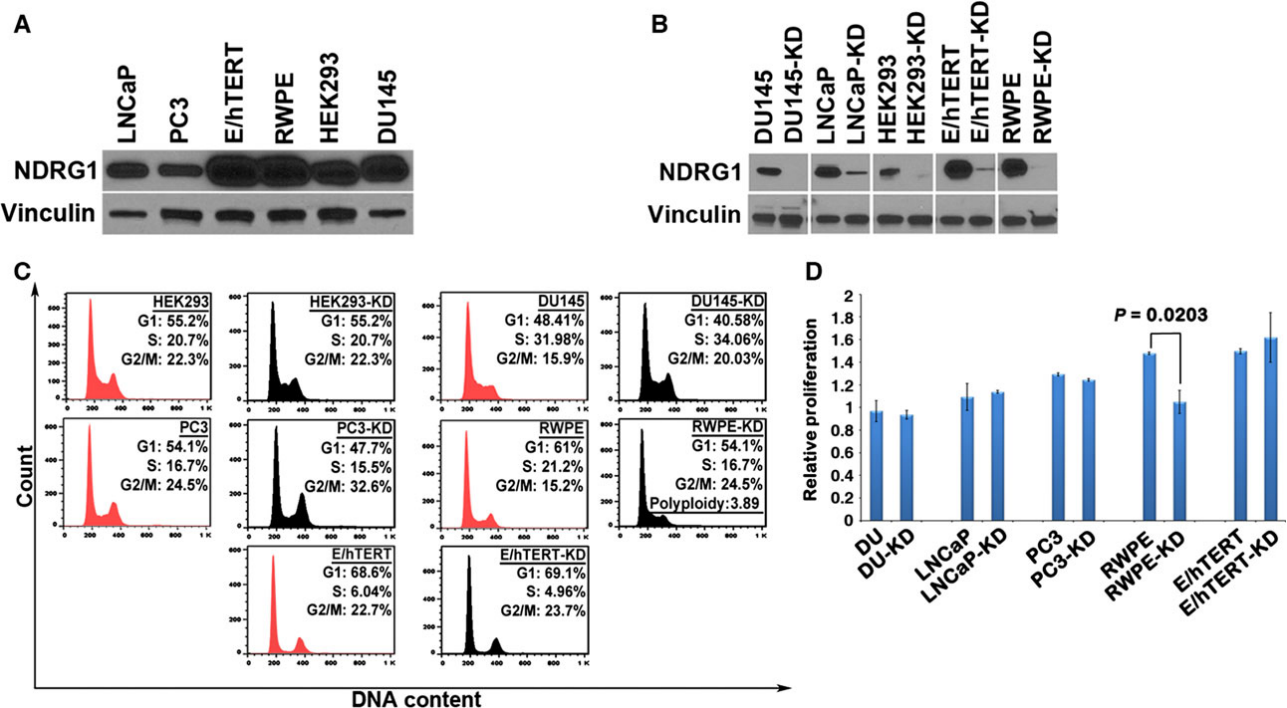
Knockdown of *NDRG1* does not affect cell cycle. (A) Representative western blot depicting protein expression of NDRG1 in HEK293 and various prostate cell lines. Vinculin is used as a loading control. (B) Representative western blot shows robust knockdown of *NDRG1* using stable lentiviral shRNA in various cell lines. Vinculin is used as a loading control. (C) Cell cycle analysis performed in *NDRG1*‐knockdown cells demonstrates no significant changes in cell cycle parameters, except in RWPE cells that show an increase in polyploidy. All western blots were repeated at least three times, and the cell cycle analysis is a representative of two independent experiments. (D) Relative proliferation of parental prostate lines and *NDRG1*‐knockdown counterparts evaluated using MTT proliferation assay. NDRG1‐knockdown cells demonstrate no significant changes in proliferation, except that RWPE‐knockdown cells show a decrease in proliferation as compared to parental cells.

**Figure 2:**
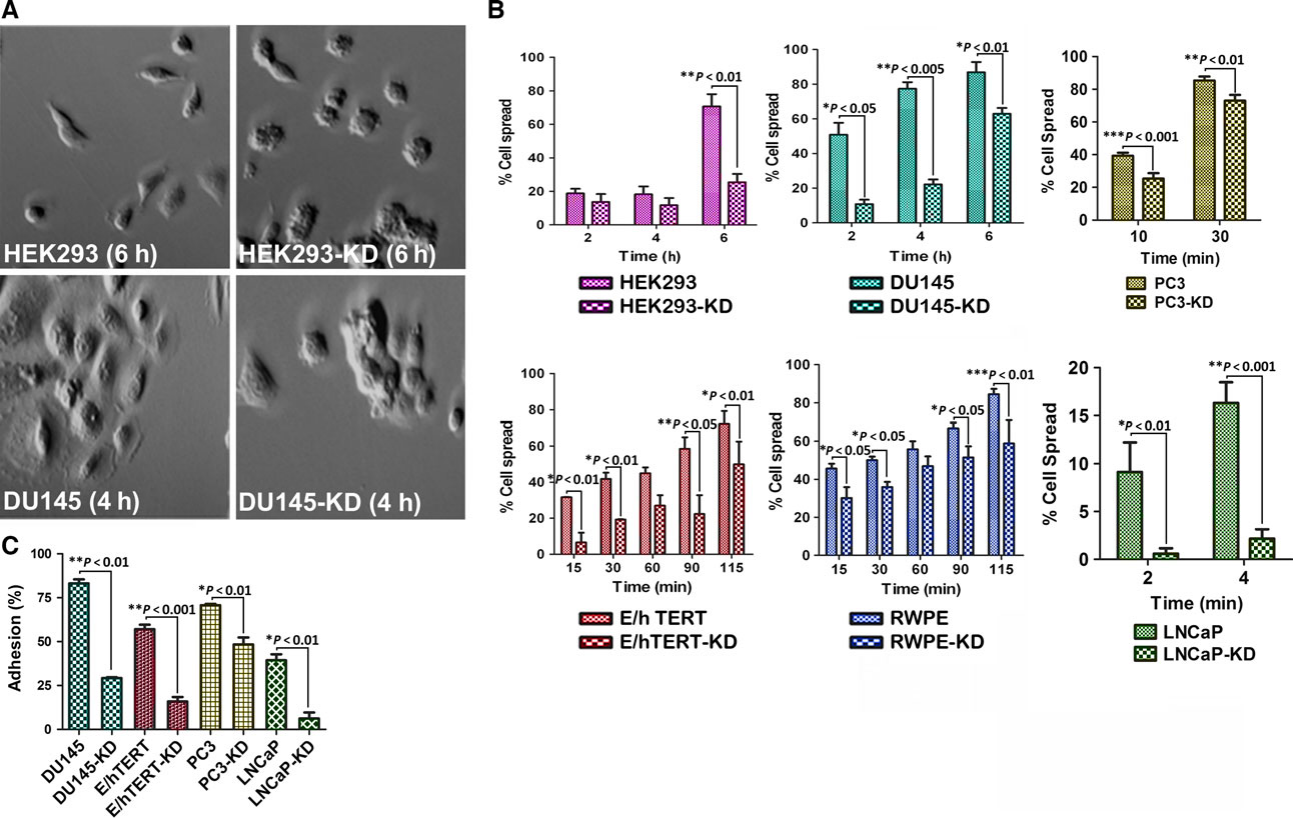
N‐myc downregulated gene 1 regulates cell spreading and cell adhesion to ECM. (A) DU‐145 and HEK293 cells were plated on plates coated with fibronectin and collagen and allowed to spread. Cells were visualized by phase‐contrast microscope for their ability to spread. As seen in the photomicrograph, *NDRG1*‐knockdown cells remained rounded and had difficulties to spread. (B) Quantitation of spreading assay after various time intervals indicates that *NDRG1* knockdown results in decreased ability of the cells to spread. (C) Adhesion assay demonstrates decrease in adhesive capacity of *NDRG1*‐knockdown cells. All experiments were repeated at least three times.

To study the behavior of cells deficient in NDRG1, we first examined the expression of NDRG1 in a panel of prostate cancer cell lines (DU145, LNCaP, and PC3 cells), normal immortalized prostate cells (RWPE and 957E/hTERT cells), and nontumorigenic HEK293 cells. While NDRG1 was expressed in all cell types examined, normal prostate epithelial cells showed the maximum expression of NDRG1 compared to other cell types (Fig. 1A). To investigate the effect of NDRG1 loss in prostate cancer cells, we generated stable knockdown of *NDRG1* using Mission Lentiviral System (Sigma) as described in our previous reports (Wissing *et al*., 2013). This system generated robust *NDRG1* knockdown, as evaluated through western blots (Fig. 1B). We next determined whether loss of NDRG1 leads to any changes in cell cycle. While there were no apparent changes in the cell cycle phases between parental and knockdown cells, *NDRG1* knockdown in RWPE cells demonstrated an increase in the number of polyploid cells (Fig. 1C). We also noted a slight, but consistent, increase in G2/M phase in all the prostate cell lines. To investigate whether there is a difference in proliferation rate after *NDRG1* knockdown in the prostate lines, we performed a MTT proliferation assay. As seen in Fig. 1D, proliferation rates between the parental and knockdown prostate lines did not change, except for RWPE cells that exhibited decreased proliferation rate as compared to parental line. Morphologically, *NDRG1* knockdown in all cell types exhibited cells that did not spread well and remained more refractile as compared to flatter and well‐spread parental cells (data not shown). Intrigued by the morphological differences, we performed a cell spreading assay to quantitate whether *NDRG1* knockdown results in decreased cell spreading. Parental and knockdown cells were allowed to spread on coverslips coated with a mixture containing fibronectin and collagen. The number of cells that appeared spread after various time intervals was counted under the microscope. As shown in Fig. 2A,B, knockdown of *NDRG1* severely decreased the kinetics of spreading on ECM. Cell spreading occurs due to coordinated action of integrin‐mediated adhesion to ECM and subsequent remodeling of the actin cytoskeleton. To investigate whether knockdown of *NDRG1* affects cellular adhesion, we performed an adhesion assay on parental and *NDRG1*‐knockdown cells. Cells were plated on dishes coated with a mixture containing fibronectin and collagen and cells were allowed to adhere for 2 h. As depicted in Fig. 2C, adherence to ECM was greatly reduced in knockdown cells. These data suggest that *NDRG1*‐knockdown cells adhere poorly to ECM matrix and exhibit decreased kinetics of cell spreading.

### 3.2 NDRG1‐deficient cells have decreased integrin expression and exhibit decreased locomotion

**Figure 3:**
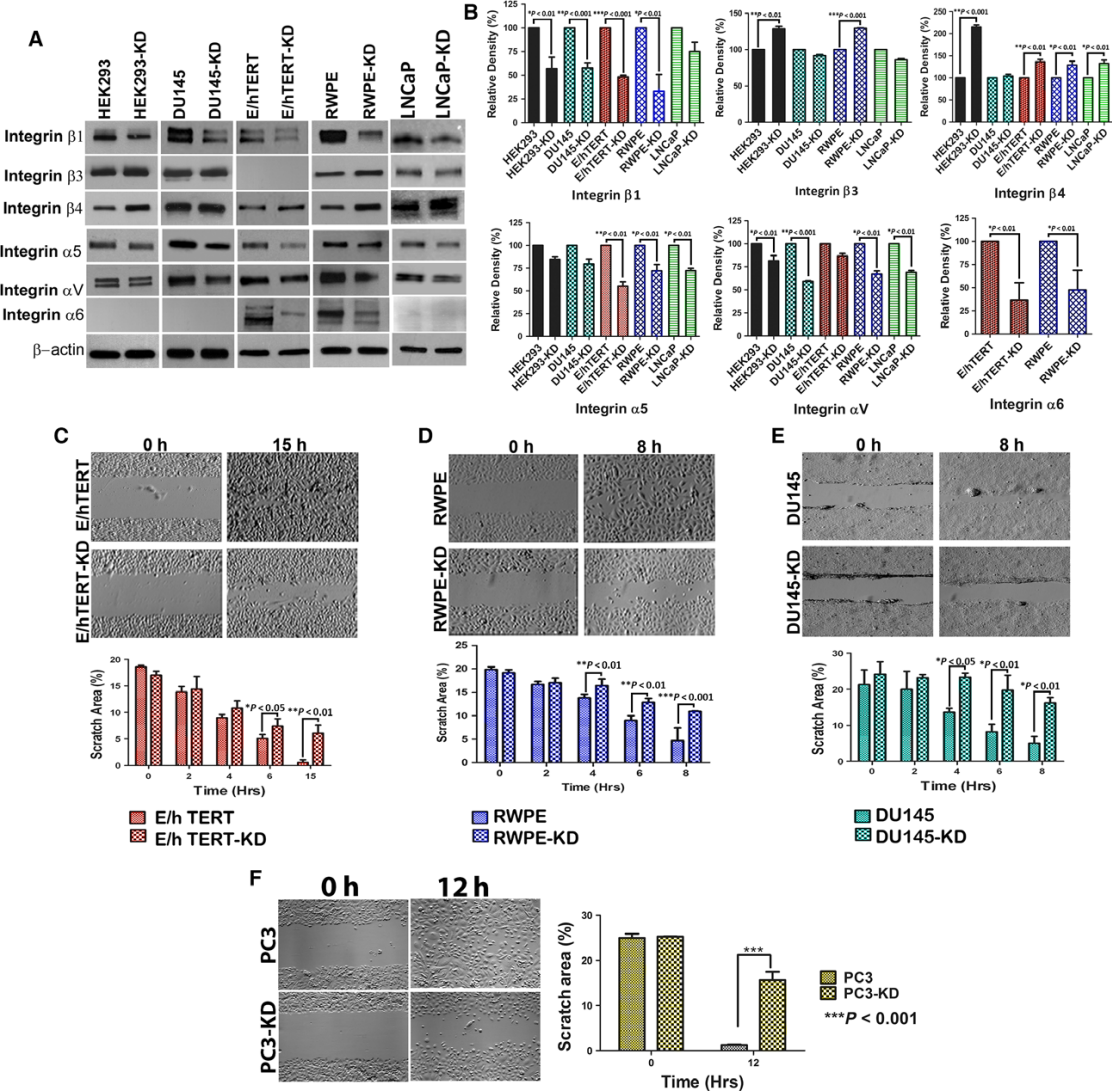
N‐myc downregulated gene 1 deficiency results in decreased protein expression of integrins and decreased cell migration. (A) Parental and knockdown cells were probed for several integrin subunits. Representative western blot demonstrates that knockdown of *NDRG1* results in decrease in several integrins. (B) Densitometric quantitation of integrin expression reveals decrease in integrin subunits in *NDRG1*‐knockdown cells. Densitometric readings were normalized to respective beta actin loading controls. (C–F) Depict wound healing assay in 957E/hTERT, RWPE, DU145, and PC3 cells, respectively. Upper panel is a representative image of wound closure after various time intervals. Lower panel is the quantitation of wound closure from three independent experiments.

As cell adhesion is mediated by integrins, we probed protein lysates from parental and knockdown cells for a panel of surface integrins. It is known that prostate cancer cells exhibit an aberrant expression of integrins (Cress *et al*., 1995; Goel *et al*., 2008). Most of the α and β integrin subunits are downregulated in prostate tumors as compared to normal prostate epithelium. Among the α subunits, α3, α4, α5, α7, and αV are downregulated, while α6 displays aberrant localization. Among the β subunits, β1_C_ and β4 are downregulated, whereas β1, β3, and β6 are upregulated (Goel *et al*., 2008). However, the status of integrin β4 remains controversial (Cress *et al*., 1995; Stewart and O'Connor, 2015; Yoshioka *et al*., 2013). *NDRG1*‐knockdown cells exhibited a marked decrease in integrin β1, α5, and αV across all the cell lines examined. Integrin β4, on the other hand, was increased in all the cell lines except DU145 cells. While integrin β3 was either not changed (in DU145) or showed a slight downregulation in LNCaP cells and an upregulation in HEK293 and RWPE cells, 957E/hTERT either did not express β3 subunit or expressed levels beyond our limits of detection (Fig. 3A,B). Similarly, DU145, LNCaP, and HEK293 cells either do not express integrin α6 or expressed it at levels beyond our detection limits. In both RWPE and 957E/hTERT cells that express integrin α6, expression was markedly downregulated in *NDRG1*‐knockdown cells. Cell adhesion is intricately linked to cell migration. The unexpected finding of decreased adhesion and cell spreading in NDRG1‐deficient cells led us to speculate that these cells would exhibit decreased cell migration. To test our hypothesis, we performed a wound healing assay on nearly confluent cultures of parental and knockdown cells. Depending on the migratory behavior of cells, we followed each cell type for various lengths of time. As predicted, *NDRG1*‐knockdown cells exhibited decreased cell migration (Fig. 3C–F). Further, live cell imaging in RWPE cells conclusively demonstrated that *NDRG1*‐knockdown cells are less migratory as compared to parental cells (Movie S1). While parental cells exhibited adhesion and retraction typical of mammalian cell locomotion, movement in *NDRG1*‐knockdown cells was restricted to filopodial extensions that appeared to form and retract back. The above observations suggest that NDRG1‐deficient cells have decreased expression of several integrins leading to decreased adhesion to the ECM and decreased locomotion. Further, because integrins signal downstream effectors that modulate actin cytoskeleton, we next determined the effect of decreased cell adhesion on molecules that regulate the actin dynamics.

### 3.3 NDRG1 modulates actin cytoskeleton by differentially affecting activities of Rho GTPases

**Figure 4:**
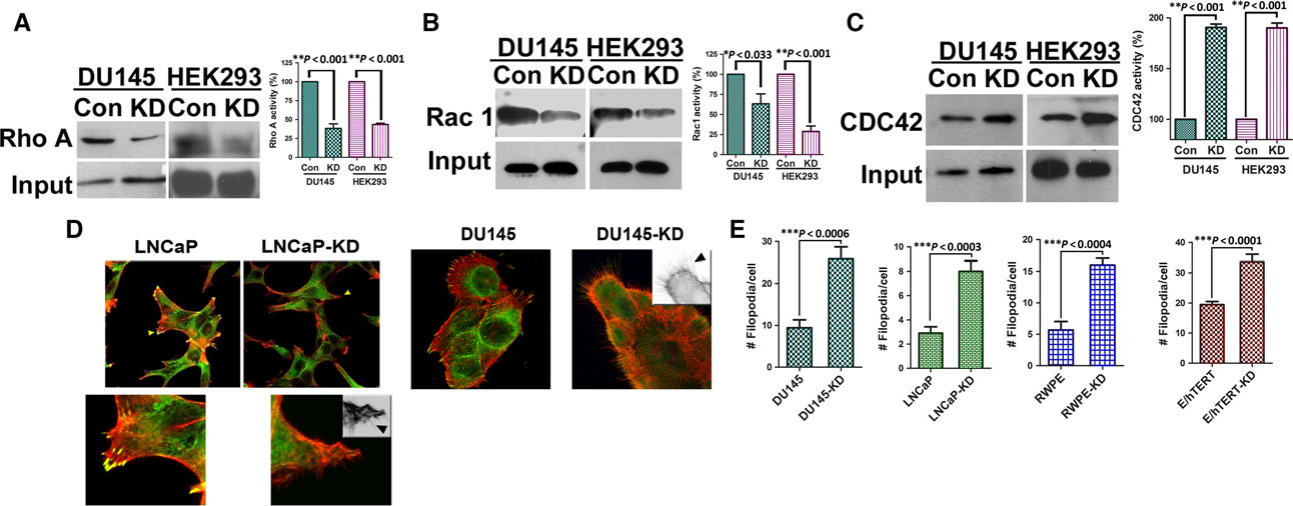
N‐myc downregulated gene 1 differentially regulates activities of Rho GTPases. A, Detergent‐solubilized extracts from HEK293 and DU145 were incubated with immobilized GST‐RBD beads, to pull down active RhoA, followed by immunoblotting with a RhoA antibody. B, Detergent‐solubilized extracts from HEK293 and DU145 were incubated with immobilized GST‐PBD beads, to pull down active Rac1 and Cdc42, followed by immunoblotting with a Rac1 antibody. C, Detergent‐solubilized extracts from HEK293 and DU145 were incubated with immobilized GST‐PBD beads, to pull down active Rac1 and Cdc42, followed by immunoblotting with a Cdc42 antibody. Each experiment was repeated three times and the amounts of GTP‐RhoA/Rac1/CDC42 were analyzed by densitometer. Left panel in A, B, and C shows representative blot of the GTP‐RhoA/Rac1/CDC42 activity assay. Right panel shows quantification and statistical analysis of the GTP‐RhoA/Rac1/CDC42. ^∗^p < 0.05. (D) Representative confocal images for immunofluorescence staining of *NDRG1*‐knockdown (LNCaP and DU145) and control cells stained with vinculin (green) and rhodamine‐conjugated phalloidin (red) to stain the focal adhesion and actin stress fibers, respectively. Magnified view of LNCaP cells shown in lower panel. Insets are inverted images for better visualization of filopodial extensions (arrow heads). *NDRG1* knockdown increases filopodial extensions. (E) Quantification of filopodial numbers in *NDRG1*‐knockdown and parental cell lines.

The ‘outside‐in’ signaling by integrins is relayed by Rho subfamily of GTPases to effectors that regulate actin polymerization leading to cell spreading and migration (Miranti and Brugge, 2002). In general, RhoA regulates actin stress fiber formation; Rac1 regulates lamellipodia formation, while Cdc42 is involved in the formation of actin‐rich protrusions called filopodia which play a role in environment sensing and cell invasion (Sahai and Marshall, 2002). We first determined the activity of each of these Rho GTPases in parental and knockdown cells using pull‐down assays. Knockdown of *NDRG1* greatly reduced Rac1 and RhoA activity, while Cdc42 was highly upregulated in the cell lines examined (Figs 4A–C and S1). Although unexpected, this observation was not surprising as Cdc42 can be activated independent of integrin signaling (Valtcheva *et al*., 2013). As Cdc42 regulates the formation of filopodia, we next visualized filamentous actin by staining formaldehyde‐fixed cells with rhodamine‐labeled phalloidin. As expected, we found an increase in filopodial extensions in *NDRG1*‐knockdown cell lines (Figs 4D,E, and S2A,B). These observations suggest that knockdown of *NDRG1* results in differential activation of Cdc42.

**Figure 5:**
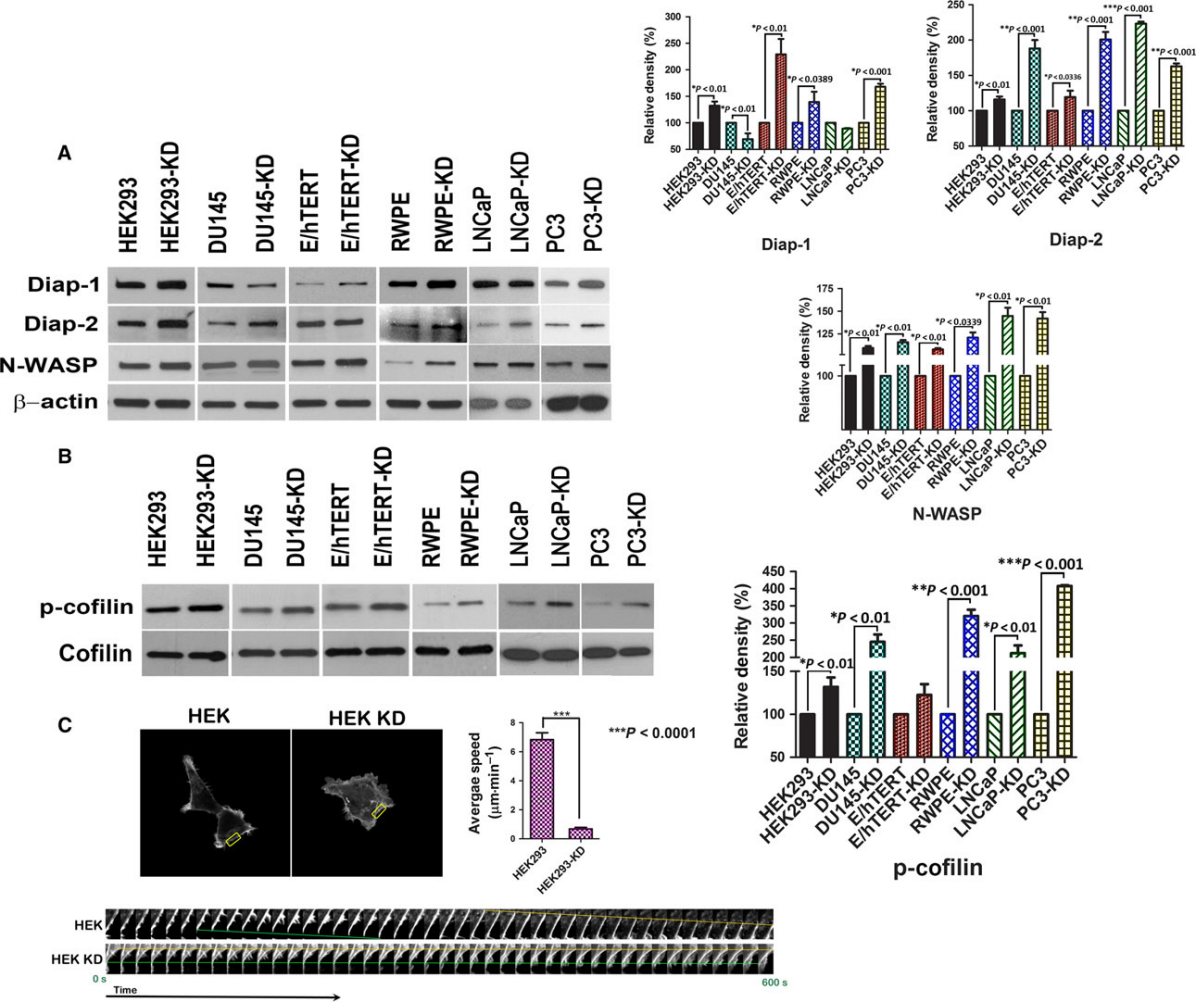
N‐myc downregulated gene 1 loss limits actin dynamics. A, Western blot for actin‐regulating proteins reveals an increase in Diap‐1, Diap‐2, and N‐WASP protein in knockdown cells. Graph on the right represents quantitative densitometric estimation of the above proteins. B, Western blot for phosphorylated cofilin after *NDRG1* knockdown reveals an increase in cofilin phosphorylation in knockdown cells without a change in total cofilin protein expression. Graph on the right depicts densitometric quantitation of phosphorylated cofilin relative to total cofilin expression. C, Parental and knockdown HEK293 cells were stably transfected with Lifeact‐RFP. Cells were plated on glass bottom dishes and viewed under Zeiss LSM780 live cell confocal microscope. Motion of cells was recorded and time frames of moving edges (boxed region) for each cell type were utilized to generate kymographs (shown below). Average speed of each cell type was quantitated and graphed.

We next investigated whether activation of Cdc42 in the knockdown cells leads to any changes in the downstream effector proteins that regulate actin dynamics. Actin dynamics involves a coordinated action of proteins that nucleate actin polymerization, proteins that stabilize newly formed actin filaments, and proteins that recycle actin monomer by severing existing actin filaments (Nurnberg *et al*., 2011). Cdc42 activates the actin nucleating Arp2/3 complex via WASP proteins. In response to Cdc42 activation, formins Diap‐1 and 2, in conjunction with N‐Wasp, positively regulate the formation of filopodia. Among the formins, Diap‐1 was found to be upregulated in HEK293‐, 957E/hTERT‐, RWPE‐, and PC3‐knockdown cells, whereas Diap‐2 was found to be markedly upregulated in DU145, RWPE, LNCaP, and PC3 cells (Fig. 5A). This could be due to different cell types employing different diaphanous subtypes to regulate filopodial extensions. Filopodial life cycle is regulated by the actin bundling and severing protein fascin and cofilin, respectively (Breitsprecher *et al*., 2011). Activated cofilin negatively regulates filopodia formation and is inactivated by phosphorylation for the formation of stable filopodia (Breitsprecher *et al*., 2011). We next investigated the total protein and phosphorylation status of cofilin after NDRG1 loss. In concordance with our data, we found an increase in cofilin phosphorylation in *NDRG1*‐knockdown cells (Fig. 5B). Concomitant increase in active LIMK2, a cofilin phosphorylating kinase which is activated by Cdc42 (Sumi *et al*., 1999), suggested that increase in cofilin phosphorylation may be a direct effect of increased LIMK2 activity (Fig. S3). An increase in cofilin phosphorylation in *NDRG1*‐knockdown cells also suggested a decrease in cofilin‐mediated severing of existing actin polymers leading to decreased actin dynamics. In order to validate this finding, we complemented these experiments by visualizing actin dynamics in live cell. For these sets of experiments, we utilized Lifeact/RFP fusion protein which selectively binds to polymerized actin without impairing actin dynamics (Riedl *et al*., 2008). Parental and knockdown HEK293 cells were stably transfected with Lifeact/RFP constructs and visualized by live cell confocal microscopy. Our data revealed that parental cells have highly dynamic actin resulting in rapid polymerization at the leading edge and depolymerization at the retracting end. In contrast, polymerized actin in the knockdown cells was restricted to filopodial extensions that formed and retracted back to the cell body (Movie S2). We quantitated the speed at which the leading edge moves in the two cell types; knockdown cells exhibited a marked decrease in speed as compared to parental cells (Fig. 5C and Movie S3). These data suggest that increased activation of Cdc42 in *NDRG1*‐knockdown cells results in limited actin dynamics which is restricted to the formation of filopodial structures at the expense of cellular locomotion.

### 3.4 NDRG1 inhibits resistance to anoikis

**Figure 6:**
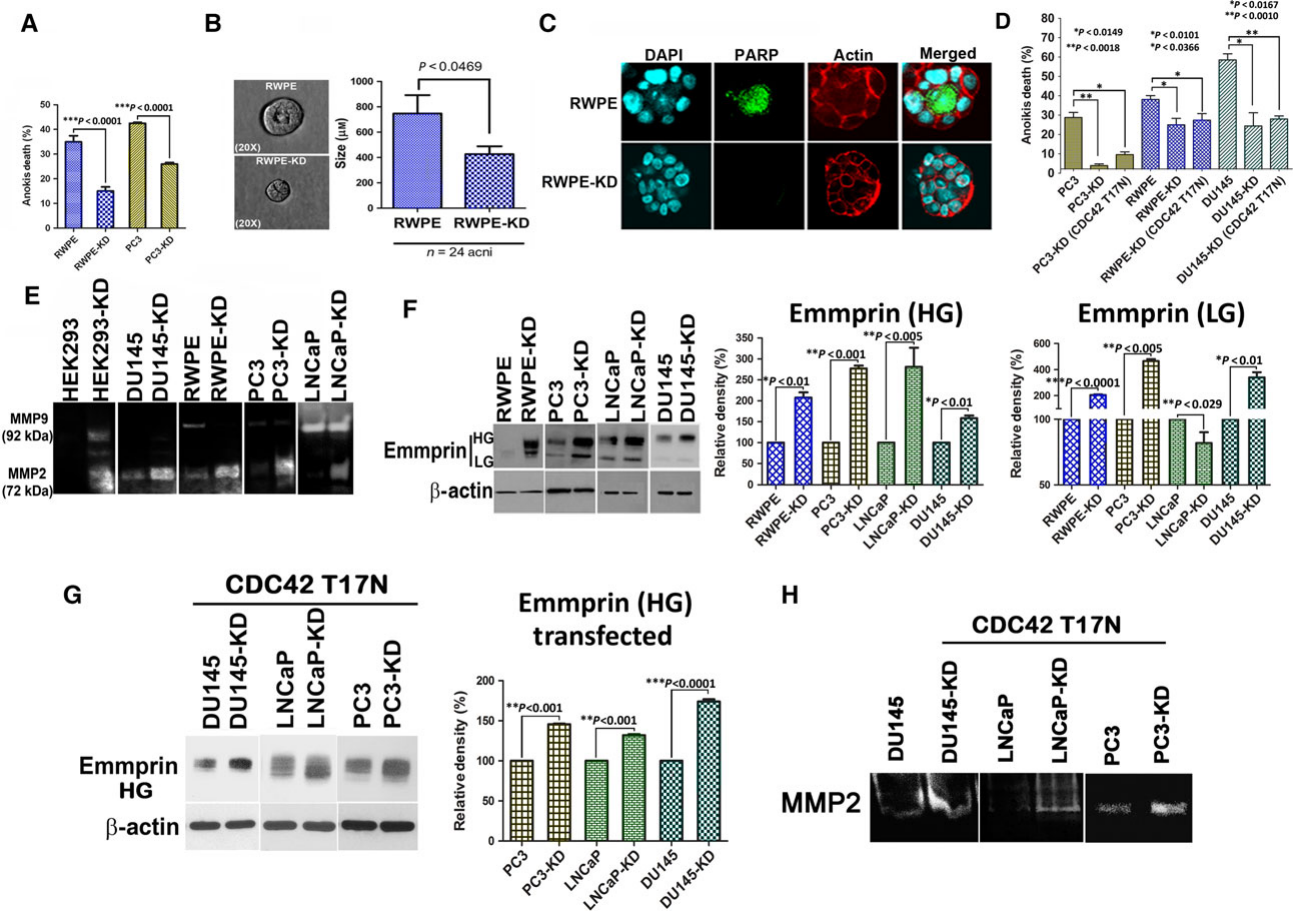
N‐myc downregulated gene 1 inhibits resistance to anoikis and matrix metalloprotease activity. (A) Parental and *NDRG1*‐knockdown cells were plated on low‐adherent dishes for 72 h, and cell death was measured by trypan blue exclusion assay. Knockdown of *NDRG1* results in decrease in anoikis‐mediated cell death. (B) Parental and knockdown RWPE cells were plated on Matrigel and allowed to divide and form acini. Phase‐contrast microscopic image shows parental and knockdown acini. Graph on the right depicts an average diameter of acini from at least three independent experiments. (C) Acini were stained for cleaved PARP (green) and actin (phalloidin, red). Cells in the center of the acini undergo anoikis‐mediated apoptosis in parental cells but knockdown cells are resistant to apoptosis. *NDRG1*‐knockdown cells had significantly decreased acini size. (D) Knockdown cells were transfected with Cdc42T17N constructs. Control knockdown and parental cells were transfected with EGFP control vectors. Anoikis assay was performed 24 h post‐transfection as described above. (E) Representative zymogram indicates that knockdown of *NDRG1* increases MMP2 and MMP9 matrix metalloprotease activities. (F) Western blot of parental and *NDRG1*‐knockdown cells for total EMMPRIN (LG, low glycosylated; HG, high glycosylated) reveals an increase in EMMPRIN expression and glycosylation in prostate cancer cells lines after *NDRG1* knockdown. Graph shows densitometric quantitation of glycosylated EMMPRIN from three different experiments. (G) *NDRG1*‐knockdown and parental prostate cancer cell lines were transfected with Cdc42T17N constructs. Forty‐eight hour post‐transfection, cell lysates from transfected cells were subjected to western blots and probed for EMMPRIN. (H) *NDRG1*‐knockdown and parental prostate cancer cell lines were transfected with Cdc42T17N constructs. Forty‐eight hour post‐transfection, media were changed to serum‐free media and secreted MMPs were analyzed by zymogram analysis. Band shows representative MMP2 activity.

Anchorage‐independent cells typically undergo a form of programmed cell death called anoikis. Increased Cdc42 activity positively impacts metastasis outcome by imparting resistance to anoikis and by increasing tumor invasiveness (Qiu *et al*., 1997). As knockdown of *NDRG1* leads to an increase in Cdc42 activity, we first investigated whether these cells are resistant to anoikis. We performed an anoikis assay by plating normal immortalized prostate epithelial cell line (RWPE) and a prostate cancer cell lines (PC3 cells) on low‐adherent dishes. Viability of the cells was monitored after 72 h, and percentage of death due to anoikis was calculated. As predicted, *NDRG1*‐knockdown cells were significantly resistant to anoikis as compared to parental cells (Fig. 6A). We complemented these experiments by testing anoikis through an acini forming assay (Debnath *et al*., 2002). In this assay, normal immortalized breast and prostate cells when grown under Matrigel undergo glandular morphogenesis and form acini. The outer layer of cells in the acini remains viable as they can adhere to the surrounding matrix; however, the inner mass of cells that are unable to form contact with the matrix undergo anoikis‐mediated apoptosis. As RWPE cells form acini and undergo glandular morphogenesis under three‐dimensional conditions (Webber *et al*., 1997), we investigated whether they can undergo anoikis after *NDRG1* knockdown. Our data indicate that acini of knockdown cells were distinctly smaller than their parental counterpart (Fig. 6B). Cells in the inner mass of *NDRG1*‐knockdown acini were resistant to anoikis and did not stain for cleaved PARP (Fig. 6C). To investigate whether increased Cdc42 activity is responsible for observed increase in anoikis resistance of NDRG1‐deficient cells, we transiently overexpressed a dominant‐negative Cdc42T17N mutant in the cells. The Cdc42 (T17N) mutant suppresses the protein's affinity for GTP and reduces its affinity for GDP and essentially is locked in an inactive state. Repeating the anoikis assay did not rescue the resistant phenotype observed in NDRG1‐deficient cells (Fig. 6D). This suggests that anoikis resistance seen in *NDRG1*‐knockdown cell lines is independent of Cdc42 activity. Altogether, these data suggest that loss of NDRG1 in prostate tumor cells may impart them with the ability to survive anoikis, consequently increasing the metastatic outcome.

### 3.5 NDRG1 regulates EMMPRIN and inhibits prostate cancer invasiveness and metastasis

**Figure 7:**
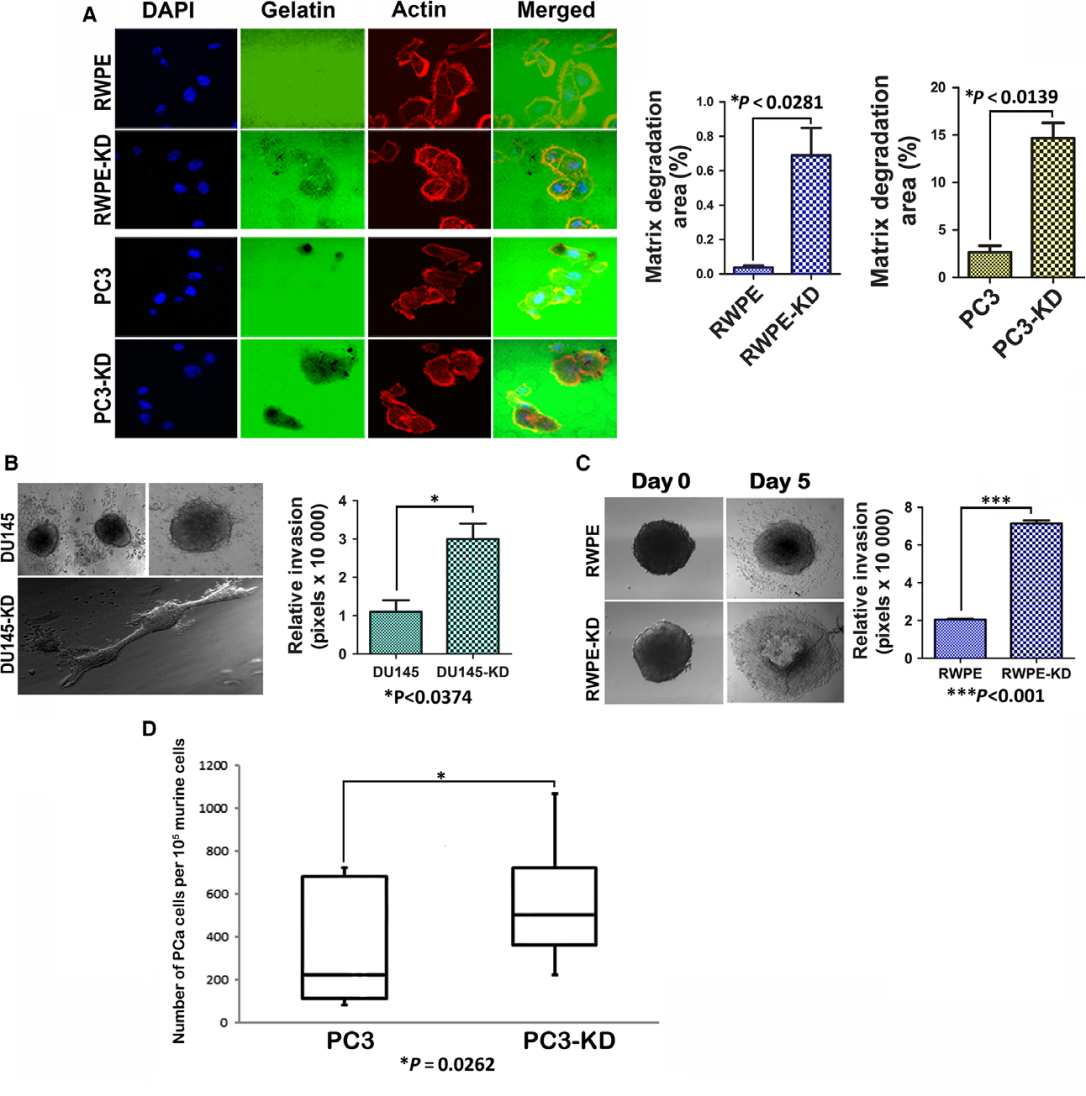
N‐myc downregulated gene 1 inhibits invadopodial activity, tumor invasion, and tumor dissemination of prostate cancer cells. (A) Parental and NDRG1‐knockdown cells were plated on FITC‐conjugated gelatin plates. Cells were fixed and stained with phalloidin and invadopodia were visualized by confocal microscopy. Knockdown of *NDRG1* significantly increased invadopodial activity in PC3 and RWPE cells. Graph depicts quantitation of invadopodial activity in the two cell types. (B and C) Three‐dimensional invasion assays on collagen matrix indicate that knockdown of *NDRG1* increases invasion index of DU145 and RWPE cells. (D) Quantitation of disseminated tumor cells in bone marrows of NOD SCID mice harboring parental and *NDRG1*‐knockdown PC3 xenografts indicates an increase in the number of disseminated tumor cells after *NDRG1* knockdown.

As an increase in Cdc42 activity positively correlates with invasive behavior of tumors (Zhang *et al*., 2010), we next evaluated whether *NDRG1* knockdown results in increased invasiveness. Matrix metalloproteases (MMPs) play a key role in invasion and their expression and activity correlate with metastasis in several cancer types, including those of the prostate (Lynch *et al*., 2005; Stearns and Stearns, 1996). Increase in Cdc42 has been shown to increase MMP activity in prostate cancer cells (Zhang *et al*., 2010). To assess MMP activity in *NDRG1*‐knockdown cells, we performed a gelatin zymogram assay. Conditioned medium from parental and knockdown cells was analyzed for MMP activity. Knockdown of *NDRG1* increased MMP9 and MMP2 activities in HEK293 cells, while DU145, RWPE, PC3, and LNCaP cells had an increase in MMP2 activity (Fig. 6E). Intrigued by a marked increase in MMP activity after *NDRG1* knockdown in every cell line that we evaluated, we wondered whether there was a general dysregulation of MMP activity in *NDRG1*‐knockdown cells. It is known that MMPs are induced by EMMPRIN, which acts as a master regulator of MMP activity in tumor cells and associated fibroblast (Biswas, 1982; Caudroy *et al*., 2002; Tang *et al*., 2004). Increased expression and glycosylation of EMMPRIN positively correlate with metastasis in several cancer types including prostate cancer (Egawa *et al*., 2006; Koga *et al*., 2007; Riethdorf *et al*., 2006; Zhu *et al*., 2012). We utilized an antibody that recognizes unglycosylated as well as both the low and heavy glycosylated forms of EMMPRIN. Immunoblots revealed that EMMPRIN expression and heavily glycosylated form were increased in the knockdown cells (Fig. 6F). Immunofluorescence analysis in RWPE cells revealed that surface EMMPRIN expression was markedly increased after *NDRG1* knockdown (Fig. S4). To assess whether increased Cdc42 activity in *NDRG1*‐knockdown cells contributes to increased EMMPRIN and MMP activities, we transfected the cells with *Cdc42T17N* mutants and assessed EMMPRIN levels in conjunction with zymogram assays. Interestingly, expression of Cdc42T17N did not alter EMMPRIN levels (Fig. 6G) or MMP activity (Fig. 6H) in knockdown cells, suggesting that the observed effects are independent of Cdc42 activity. As increased glycosylation of EMMPRIN and MMP activities positively correlate with an increase in invadopodia formation (Grass *et al*., 2012; Hu *et al*., 2011), we performed an invadopodia assay on RWPE and PC3 cells by plating cells on fluorescent gelatin‐coated coverslips. Direct visualization of invadopodia‐mediated matrix degradation was performed by confocal microscopy. As seen in Fig. 7A, under our experimental condition, normal immortalized prostate epithelial RWPE parental cells did not have noticeable invadopodial activity, while parental PC3 cells did have higher invadopodia formation than RWPE cells. However, *NDRG1* knockdown in both the cell types significantly increased invadopodia formation. This indicates that loss of NDRG1 results in increase in prostate cancer invasion. To validate and quantitate invasion in *NDRG1*‐knockdown cells, we utilized a three‐dimensional invasion assay, which is more close to *in vivo* settings than the Boyden chamber invasion assay. Prostate cancer cell lines and HEK293 cells were grown as hanging drop to form spheroids. Spheroids were embedded in either collagen IV or Matrigel matrices, and invasion was monitored daily through phase‐contrast microscope to determine invasion. HEK293 cells do not invade in collagen matrix; however, when placed in Matrigel, HEK293 NDRG1‐knockdown cells exhibited actin‐rich microextensions throughout the surface of the spheroids (Fig. S5A). Protrusions from HEK293 NDRG1‐knockdown cells extended out into the matrix and increased as a function of time (Fig. S5B). This collective invasive behavior was either not observed (HEK293) or was much muted (DU145 and RWPE) in parental cells. *NDRG1*‐knockdown cells had a significantly increased invasion index when compared to parental cells (Fig. 7B,C). PC3 and LNCaP cells could not be utilized for the spheroid assay as PC3 cells formed loose spheroids that disintegrated to individual cells on collagen matrix and LNCaP cells developed necrotic centers that made assessment of invasion technically challenging (Fig. 5C). Whether cellular invasiveness observed after *NDRG1* knockdown leads to an increase in disseminated prostate cancer cells was the next question we addressed. Subcutaneous xenografts from parental and *NDRG1*‐knockdown PC3 cells were established in NOD SCID mice using our previously described protocols for metastasis assay (Havens *et al*., 2008; Shiozawa *et al*., 2011). After three weeks, mice tibias were assessed for bone metastasis. Disseminated cancer cells from tumor xenografts can be quantitated by real‐time PCR (Alcoser *et al*., 2011; Havens *et al*., 2008; Shiozawa *et al*., 2011). As shown in Fig. 7D, the number of disseminated tumor cells in knockdown‐bearing mice was significantly higher than in mice that harbored parental PC3 tumors. This indicated that NDRG1‐deficient PC3 tumors are more invasive as compared to their parental counterparts.

## 4 Discussion

Metastasis is a very inefficient process with only a fraction of total cells that seed distant organs demonstrating metastatic growth. Metastasis suppressors, by definition, are genes that prevent metastasis without preventing tumorigenicity. Insights into the molecular function of metastasis suppressors could aid in understanding pathways/barriers that prevent the process of metastasis. Of the handful of metastasis suppressors in prostate cancer, *NDRG1* has been a relatively recent addition (Bandyopadhyay *et al*., 2003, 2004). Although the clinical status of *NDRG1* is well documented, mechanisms by which NDRG1 prevents metastasis remain poorly understood. We and others have shown that NDRG1 may function as a vesicular transport protein (Askautrud *et al*., 2014; Kachhap *et al*., 2007b; Pietiainen *et al*., 2013). In the present study, we provide molecular insights into the mechanism by which NDRG1 prevents prostate metastasis. We utilized a panel of prostate cancer cell lines (androgen receptor positive (LNCaP), androgen receptor negative (DU‐145 and PC3)), noncancerous immortalized prostate cell lines (RWPE‐1 and 957E/hTERT), and a noncancerous immortalized nonprostate line (HEK293) to understand the molecular pathways that NDRG1 regulates to elicit its metastasis suppressor function. Adhering to the dogma that the loss of metastasis suppressors generally does not lead to an increase in proliferation, our results indicate that except for RWPE cells that show decreased cell proliferation after knockdown, loss of NDRG1 does not lead to significant changes in cell cycle. It is likely that the low proliferation rate in RWPE cells could be due to centrosomal defect after *NDRG1* knockdown as reported (Croessmann *et al*., 2015). Intriguingly, certain phenotypes associated with metastasis such as matrix adhesion, cell spreading, and motility, which otherwise have been shown to increase after loss of metastasis suppressors (Nguyen *et al*., 2009), were severely hampered in prostate cancer cells that were deficient in NDRG1. In this respect, *NDRG1* differs from known metastasis suppressors and from a recent observation that suggested an increase in motility of DU‐145 cells after NDRG1 loss (Jin *et al*., 2014). However, Li et. al. utilized a single prostate cancer line in their study. One experimental difference between their study and ours is that the previous study was based on transient knockdown of *NDRG1*, while our results are derived from stable *NDRG1* knockdowns in multiple cell lines. The observed characteristics of *NDRG1*‐knockdown cells in our study, at least in part, can be explained by loss of expression of several integrins in *NDRG1*‐knockdown cells, consequently leading to decrease in activities of downstream RhoA and Rac1 GTPases that regulate actin dynamics. An increase in Cdc42 activity observed after NDRG1 loss is likely to be integrin independent (Valtcheva *et al*., 2013). Although our work investigated three major Rho GTPases that modulate actin cytoskeleton, we cannot rule out the likelihood of other Rho GTPase being deregulated in *NDRG1*‐knockdown cells. An increase in RhoC GTPase was shown to increase the invasion of PC3 prostate cancer cells without affecting the motility (Yao *et al*., 2006). Nonetheless, our work clearly demonstrates that NDRG1‐deficient cells have an elevated Cdc42 activity leading to increase in filopodial actin. Intriguingly, all the NDRG1‐deficient cell lines that we examined exhibited decreased actin dynamics and increased invasion irrespective of their tissue origin or neoplastic properties. Thus, it appears as if NDRG1 functions in a general pathway that regulates actin dynamics and cellular invasiveness. Prostate cancer cells deregulate this pathway by downregulating NDRG1 to invasive property. A novel insight from our study is the general deregulation of MMPs and its inducer EMMPRIN. Our findings suggest that loss of NDRG1 increases highly glycosylated form of EMMPRIN. This is particularly interesting, as increased glycosylation of EMMPRIN is found in many cancers including those of prostate (Papadimitropoulou and Mamalaki, 2013; Riethdorf *et al*., 2006). Understanding how NDRG1 regulates EMMPRIN surface expression and glycosylation would provide further insights into the metastasis suppressor function of NDRG1. Targeting EMMPRIN/MMPs axis in NDRG1‐deficient prostate tumors could be an attractive therapeutic option to decrease prostate cancer metastasis. In summary, our findings indicate that loss of NDRG1 decreases actin dynamics resulting in decreased cellular migration. However, increased Cdc42 activity after loss of NDRG1 imparts a highly invasive phenotype which is also mediated by increased surface expression of EMMPRIN and deregulated MMP activities.

## Author contributions

SK, AS, and JM conceived and designed the project. AS, JM, JY, HK, JEV, and JEZ acquired the data. SK, MC, HH, and KJP analyzed and interpreted the data. AS, JM, and SK wrote the manuscript.

## Supporting information


**Fig. S1.** Knockdown of NDRG1 differentially regulates Rho GTPases in LNCaP cells.

**Fig. S2.** Knockdown of NDRG1 increases filopodia in normal immortalized prostate cells.

**Fig. S3.** Loss of NDRG1 increases phosphorylated LIMK2.

**Fig. S4.** Loss of NDRG1 increases surface expression of EMMPRIN.

**Fig. S5.** Loss of NDRG1 increases collective migration in HEK293 cells.

**Movie S1.** Parental and NDRG1 knockdown RWPE cells were plated on 60 mm tissue culture dishes and allowed to adhere overnight.

**Movie S2.** Parental and NDRG1 knockdown HEK293 cells stably transfected with Lifeact actin probe were visualized under live cell confocal microscopy.

**Movie S3.** Leading edges of parental and NDRG1 knockdown HEK293 cells stably transfected with Lifeact actin were recorded under live cell confocal microscopy.
