# Correction to “The prostate metastasis suppressor gene NDRG1 differentially regulates cell motility and invasion”

**DOI:** 10.1002/1878-0261.13662

**Published:** 2024-05-29

Sharma A, Mendonca J, Ying J, Kim H‐S, Verdone JE, Zarif JC, Carducci M, Hammers H, Pienta KJ and Kachhap S. The prostate metastasis suppressor gene NDRG1 differentially regulates cell motility and invasion. *Mol Oncol*. 2017;11:655–669. https://doi.org/10.1002/1878-0261.12059

The article by Sharma et al. contained inadvertent duplications between western blot images presented in Fig. 3A and suboptimal contrast settings in western blot images presented in Fig. 6F. The authors have provided the original raw data for all experimental replicates and have corrected the figure panels in question. The revised figures are included here. All authors agree to this corrigendum and confirm that changes do not affect the conclusions of the article.

**Figure:**
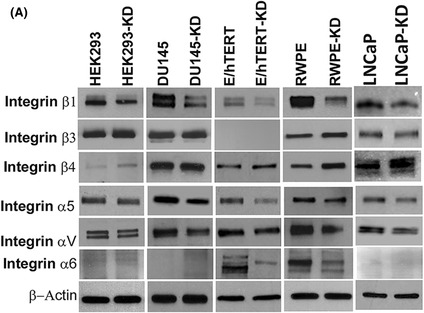
Figure 3A.

**Figure:**
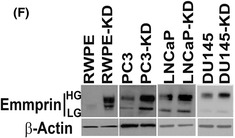
Figure 6F.

The authors apologize for any inconvenience caused.

