# Supplementary figures and images for: The prostate metastasis suppressor gene NDRG1 differentially regulates cell motility and invasion
Source: Mol Oncol. 2017 May 2;11(6):655–69. doi: 10.1002/1878-0261.12059 (PMC5467496; doi:10.1002/1878-0261.12059)

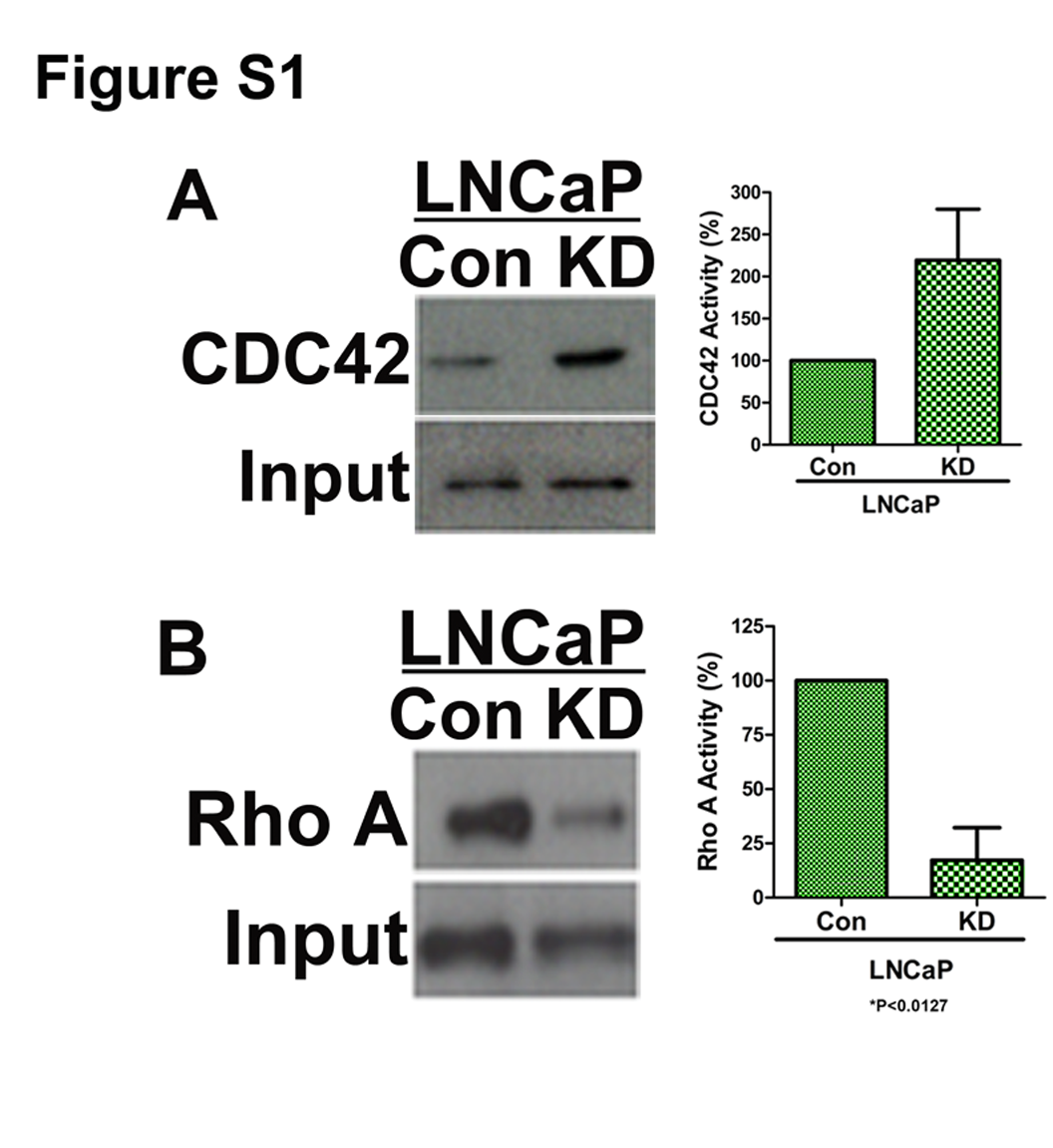

Supplement: Supplementary file 1 — Fig. S1. Knockdown of NDRG1 differentially regulates Rho GTPases in LNCaP cells. [file MOL2-11-655-s001.tif]

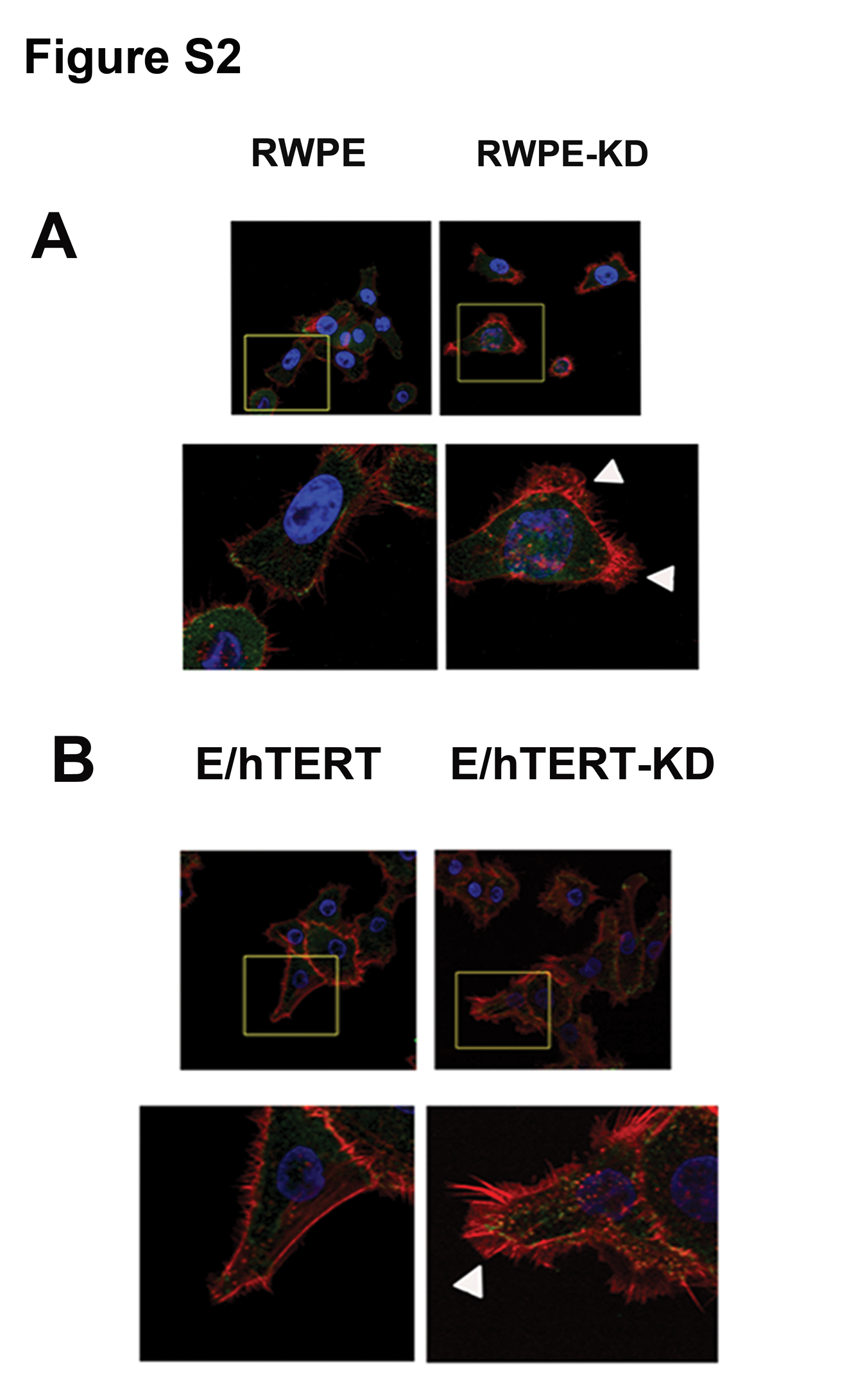

Supplement: Supplementary file 2 — Fig. S2. Knockdown of NDRG1 increases filopodia in normal immortalized prostate cells. [file MOL2-11-655-s002.tif]

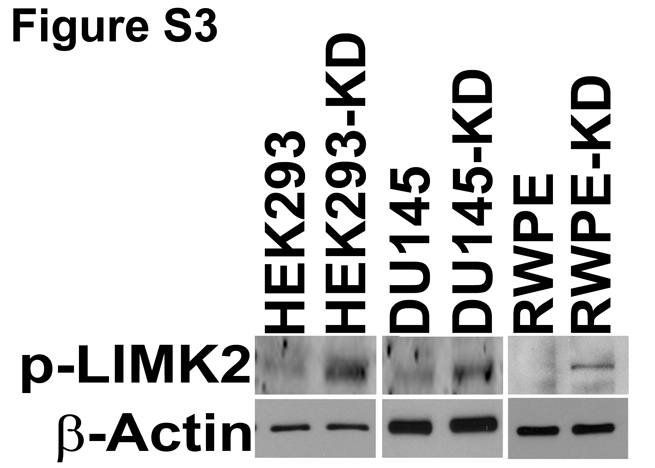

Supplement: Supplementary file 3 — Fig. S3. Loss of NDRG1 increases phosphorylated LIMK2. [file MOL2-11-655-s003.tif]

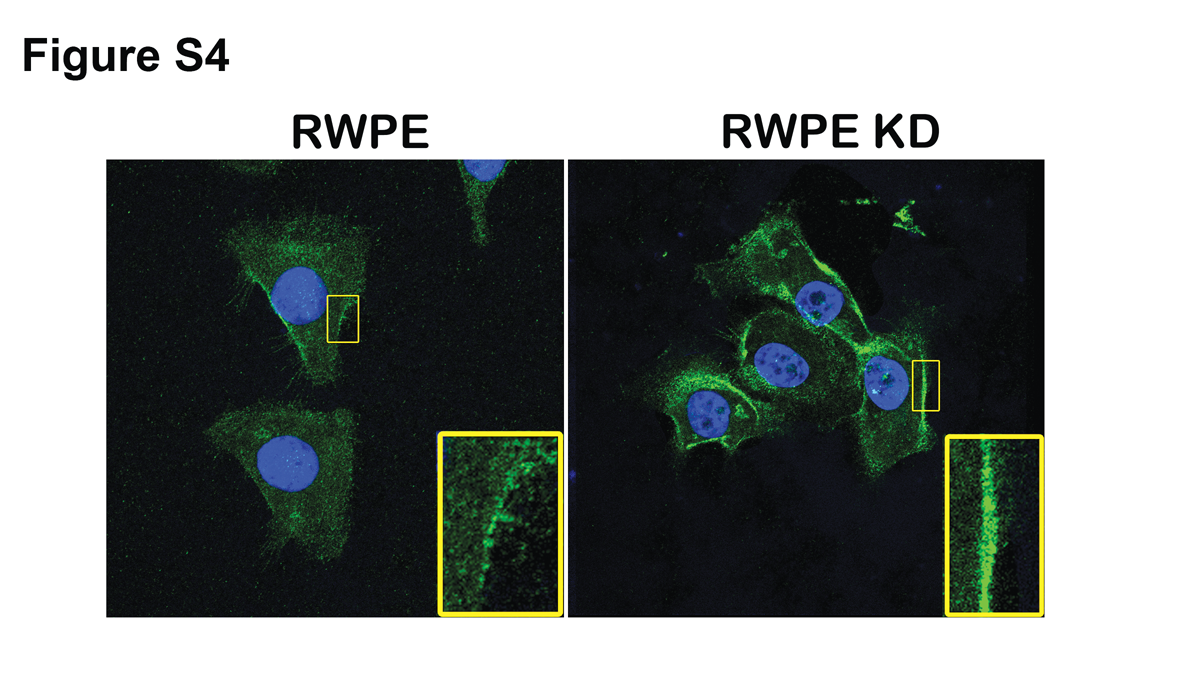

Supplement: Supplementary file 4 — Fig. S4. Loss of NDRG1 increases surface expression of EMMPRIN. [file MOL2-11-655-s004.tif]

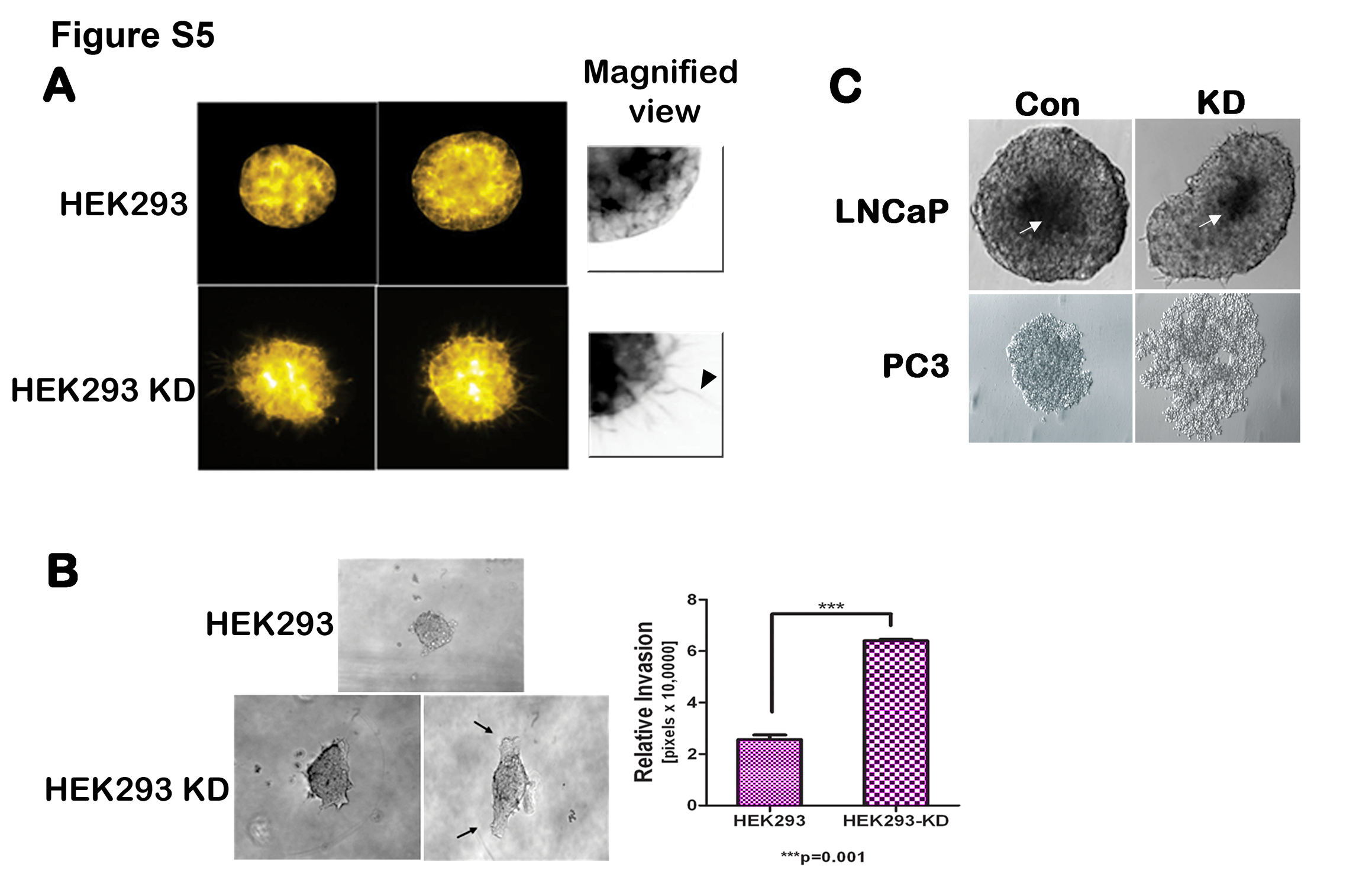

Supplement: Supplementary file 5 — Fig. S5. Loss of NDRG1 increases collective migration in HEK293 cells. [file MOL2-11-655-s005.tif]
